# Violence against children, later victimisation, and mental health: a cross-sectional study of the general Norwegian population

**DOI:** 10.3402/ejpt.v6.26259

**Published:** 2015-01-13

**Authors:** Siri Thoresen, Mia Myhre, Tore Wentzel-Larsen, Helene Flood Aakvaag, Ole Kristian Hjemdal

**Affiliations:** 1 Norwegian Centre for Violence and Traumatic Stress Studies, Oslo, Norway; 2 Department of Pediatrics, Oslo University Hospital Ullevål, Oslo, Norway; 3 Centre for Child and Adolescent Mental Health, Eastern and Southern Norway

**Keywords:** Violence, child abuse, child sexual abuse, rape, mental health, revictimisation, epidemiology, anxiety, depression

## Abstract

**Background:**

Violence in childhood is associated with mental health problems and risk of revictimisation. Less is known about the relative importance of the various types of childhood and adult victimisation for adult mental health.

**Objective:**

To estimate the associations between various types of childhood and adult violence exposure, and their combined associations to adult mental health.

**Method:**

This study was a cross-sectional telephone survey of the Norwegian adult population; 2,435 women and 2,092 men aged 18–75 participated (19.3% of those we tried to call and 42.9% of those who answered the phone). The interview comprised a broad array of violence exposure in both childhood and adulthood. Anxiety/depression was measured by the Hopkins Symptom Check List (HSCL-10).

**Results:**

Victimisation was commonly reported, for example, child sexual abuse (women: 10.2%, men: 3.5%), childhood–parental physical violence (women: 4.9%, men: 5.1%), and lifetime forcible rape (women: 9.4%, men: 1.1%). All categories of childhood violence were significantly associated with adult victimisation, with a 2.2–5.0 times higher occurrence in exposed children (p<0.05 for all associations). Anxiety/depression (HSCL-10) associated with adult abuse increased with the number of childhood violence categories experienced (p<0.001). All combinations of childhood violence were significantly associated with anxiety/depression (p<0.001 for all associations). Individuals reporting psychological violence/neglect had the highest levels of anxiety/depression.

**Conclusions:**

Results should be interpreted in light of the low response rate. Childhood violence in all its forms was a risk factor for victimisation in adulthood. Adult anxiety/depression was associated with both the number of violence categories and the type of childhood violence experienced. A broad assessment of childhood and adult violence exposure is necessary both for research and prevention purposes. Psychological violence and neglect should receive more research attention, especially in combination with other types of violence.

Childhood violence is related to mental health problems in adulthood, as demonstrated by both retrospective (Chen et al., 2010; Green et al., 2010) and prospective (Caspi et al., 2003; Noll, Horowitz, Bonanno, Trickett, & Putnam, 2003) studies. Unfortunately, violence against children is not uncommon in the general population (Briere & Elliott, 2003) and hence constitutes a public health problem. Although there is a strong relationship between childhood violence and adult mental health problems, the link is not necessarily simple or direct. Continued social deprivation, drug and alcohol use, genetic factors and changes in stress-response systems, and cognitions, resource loss, and emotions such as self-blame and shame are among factors that may represent potential mediators (Caspi et al., 2003; Fergusson, Horwood, & Lynskey, 1997; King & Liberzon, 2012; Schumm, Doane, & Hobfoll, 2012; Zayfert, 2012). Revictimisation is one factor that has received substantial empirical support as a potential pathway from childhood violence to adult psychological distress (Pratchett & Yehuda, 2011).

Research on revictimisation has traditionally been limited to child sexual abuse (CSA) and subsequent adult sexual victimisation. Some researchers even restrict their definition as such (Roodman & Clum, 2001). An increased and large revictimisation risk in CSA victims has been documented (Classen, Palesh, & Aggarwal, 2005; Messman & Long, 1996). In earlier studies, victimisation was often investigated separately for various types of violence, which resulted in parallel research on, for example, CSA and child physical maltreatment. During recent years, this research has become more integrated and has thus produced robust evidence that violence victims are often exposed to multiple types of victimisation (Finkelhor, Ormrod, & Turner, 2007). Adult health seems to be highly impacted by the cumulative burden of victimisation (Cloitre et al., 2009; Felitti et al., 1998; Zayfert, 2012). However, to date, there is no clear understanding of the relative importance of specific types of victimisation compared to the total burden of violence for adult revictimisation and mental health. Finkelhor and colleagues (2007) argue against a narrow definition that investigates only one type of victimisation at early and later time points, because one type of victimisation may also increase the later risk of other types of violence. Similarly, Teicher and colleagues (2006) note that some types of violence, such as psychological abuse, have been largely ignored. Several authors have called for a broad assessment of childhood exposure to violence to better identify young people at risk for later revictimisation and health problems (Miller et al., 2011).

To investigate the importance of various childhood and adult violence exposure for mental health, we conducted a large, cross-sectional study of violence exposure in the general Norwegian population. We used a broad assessment of childhood abuse that followed the World Health Organization's categorisation of violence into sexual, physical, psychological abuse and neglect (World Report on Violence and Health, 2002), and included adult sexual abuse, physical abuse, and intimate partner violence (IPV). We hypothesised that childhood violence exposure would increase the risk of adult violence exposure, and in addition that childhood violence exposure would increase the vulnerability for developing mental health problems following adult exposure.

The aims of the study were to: 1) estimate the association between childhood violence exposure and adult violence exposure in the general Norwegian population; 2) investigate the association between both childhood and adult violence exposure and adult mental health; and 3) investigate the importance of the various combinations of childhood violence.

## Methods

### Participants and procedure

A random sample of Norwegian citizens aged 18–75 was drawn from the General Population Registry of Norway, which contains records of all inhabitants’ personal identification number, date of birth, sex, and address. All individuals first received a postal invitation letter with information about the study, and they were subsequently phoned and asked to consent to participation in the study. Those who consented were interviewed by telephone. The only exclusion criteria were inability to participate because of language problems, difficulties in hearing, intellectual disability, or intoxication, as evaluated by the interviewer.

Altogether, 40,000 invitation letters were distributed, although not all of these individuals were contacted, and 899 individuals called or mailed to inform that they did not want to be contacted by telephone. For 7,130 individuals, no telephone number could be identified. Of the remaining 31,971, 23,441 individuals were actually called. Individuals were called randomly from the population registry sample, and calling stopped when the pre-specified sample size was achieved. The mean number of calls made to those who never answered the phone ranged from 1 to 18, with a mean of 5.6. Of these, 13,794 did not answer the phone, leaving 9,647 individuals who actually answered the phone and were asked to consent to participating. Of these, 5,120 declined participation, and 4,527 participated. Not including unidentified telephone numbers and unanswered phone calls, which is comparable to the random digit dialling procedures, the response rate was 42.9% (women: 45.0%, men: 40.8%). Compared to the rest of the sample of 40,000, responders were more often female (53.8% versus 48.9%, chi square p<0.001) and were slightly older (mean age 43.9 versus 43.2, t-test p=0.004). Compared to those who we reached by phone, but who rejected participation, responders were more often female (53.8% versus 49.6%, chi square p<0.001) and were somewhat younger (mean age 43.9 versus 46.8, t-test p<0.001).

As we did not have information on marital status, educational level, and household income for the drawn sample, we compared the respondents with corresponding population figures from Statistics Norway on these variables (http://www.ssb.no/en/statistikkbanken). Approximately equal proportions of the respondents and the population at large were married, 45.0% vs. 45.0% for women and 44.6% vs. 45.4% for men, but a significantly smaller proportion of our respondents compared to the population was divorced or separated, 11.0% vs. 14.7% for women and 8.9% vs. 10.9% for men (chi square p<0.001). Almost two times the proportion of the respondents compared to the total population had a university or college education 47.7% vs. 26.0% for men (chi square p<0.001) and 56.2% vs. 31.6% for women (chi square p<0.001). The respondents were also economically better off than the population as a whole, 49% of the respondents vs. 37% of the population reported a household income of more than € 85,725 (chi square p<0.001). These analyses of marital status, education, and household income suggest a positive selection of respondents.

However, these analyses of socio-demographic cannot tell us whether the sample is biased in the variables under investigation. It may be likely that violence-exposed individuals considered the study to be more relevant to them, and potentially made themselves more available via telephone. We performed analyses within responders of associations between number of calls required to get in touch and socio-demographic variables as well as violence exposure. Details are described in Appendix 1. These “hard to contact” analyses do not generally support the hypothesis that individuals with more exposure or more mental health problems were easier to contact. However, women who reported physical violence in childhood and men who reported emotional neglect seemed to be slightly more available. This might indicate a small overrepresentation of these types of violence.

Telephone interviews were conducted by the data collection agency Ipsos MMI from 23 April to 7 July 2013.

The structure of the telephone interview followed the design of three national studies in the USA (Kilpatrick, 2004; Resnick, Kilpatrick, Dansky, Saunders, & Best, 1993), and was expanded to include a detailed assessment of childhood violence. The questions were direct and as behaviour-specific as possible. Each affirmative answer was followed by a series of supplementary questions. Interviewers were instructed to make sure that participants had the necessary privacy during the interview to ensure their safety. At the end of the interview, participants were asked if they were distressed by the questions and needed to talk to someone (1.5%, N=66). They were subsequently referred to an external follow-up service if they so wished (0.8%, N=37). The study was approved by the Regional Committee for Medical and Health Research Ethics in South-East Norway.

## Measures

### Childhood violence

#### Child sexual abuse

Child sexual abuse was introduced with the text “Sometimes children can be tricked, rewarded or threatened to engage in sexual acts they don't understand or are unable to stop,” followed by the question: “Before you were 13 years of age, did anyone who was at least 5 years older than you have any form of sexual contact with you?” If the respondent answered affirmatively, follow-up questions asked if the sexual act included vaginal, oral or anal penetration (Kilpatrick et al., 2000, 2003). *Forcible rape* was measured by four questions introduced in The National Women's Study (Kilpatrick, Edmunds, & Seymour, 1992) and later used by the National Violence Against Women Survey (Tjaden & Thoennes, 1998): “Has anyone ever forced you into 1) intercourse, 2) oral sex, or 3) anal sex, or 4) put fingers or objects in your vagina or anus by use of physical force or by threatening to hurt you or someone close to you?” Forcible rape was defined as an affirmative response to any one of these four questions. Participants indicated their age at the time of the rape (or their age at the first and last time of rape in cases with more than one incident); this information was used to create variables defining rape before the age of 18.

#### Parental physical violence

Parental physical violence included four questions: “Have you ever been 1) hit with a fist or a hard object, 2) kicked, 3) beaten up, or 4) physically attacked in other ways?” (Kilpatrick et al., 2003). *Parental IPV* included one parent slapping, hitting with a fist or an object, kicking, strangulating, or otherwise physically attacking the other parent. *Parental psychological violence* was measured by a slightly adapted single question from the Stressful Life Events Screening Questionnaire (Goodman, Corcoran, Turner, Yuan, & Green, 1998): “Did your parent(s) repeatedly ridicule you, put you down, ignore you, or tell you that you were no good?” *Parental emotional neglect* was measured by the question: “In your childhood, how often did you feel loved?” *Parental physical neglect* was measured by the question: “In your childhood, how often did you feel that someone could take care of you and protect you?” Both neglect questions were drawn from the Adverse Childhood Experiences Study (Centers for Disease Control and Prevention, 2014). Both neglect questions were measured on a five-point scale ranging from “never” to “very often or always.” Responding “never,” “seldom,” or “sometimes” defined neglect. Parental violence included violence from biological parents or other caregivers in parental positions.

### Adult violence

*Forcible rape* in adulthood was defined as at least one affirmative answer to any of the four rape questions described above, when the participant was 18 or older for one or more occurrences. *Physical violence* at 18 or older included six questions: “Have you ever been 1) hit with a fist or a hard object, 2) kicked, 3) strangulated, 4) beaten up, 5) threatened with a weapon, and/or 6) physically attacked in other ways?” (Kilpatrick et al., 2003). Follow-up questions identified perpetrator relationships, and when the perpetrator was a partner or ex-partner, the violence was categorised as *intimate partner violence*. Participants who reported other perpetrators and in addition reported that they, during the incident, experienced fear of sustaining injury were categorised as *physical violence*. This restriction was made to ensure that minor incidents were not included. Individuals could report several perpetrators and hence could report both IPV and other physical violence.

#### Anxiety/depression

To reduce interview time, an abbreviated 10-item version of the Hopkins Symptom Checklist-25 (HSCL; Derogatis, Lipman, Rickels, Uhlenhuth, & Covi, 1974) was used in this study. Five items intended to measure last week's symptoms of depression (feeling hopeless about the future; feeling blue; blaming yourself for things; feeling everything is an effort; and feeling of worthlessness) and five items intended to measure anxiety (suddenly scared for no reason; faintness, dizziness or weakness; feeling fearful; feeling tense or keyed up; difficulties falling asleep, staying asleep). Participants responded on a scale from 0 (not bothered) to 3 (bothered a great deal). This abbreviated version of the HSCL has shown good psychometric properties, and has previously been found to correlate highly (r=0.97) with the HSCL-25 in a general population sample (Tambs & Moum, 1993). A cut-off value of >1.85 achieved the best combination of specificity, sensitivity, and predictive values (Strand, Dalgard, Tambs, & Rognerud, 1993) against the 5-items Mental Health Index (Ware, Snow, & Kosinski, 2000). In the current study, the Cronbach's alpha for the 10 items was 0.89.

Socio-demographic variables included gender, age (at the time of interview), marital status, occupational status, and education level.

### Statistical procedures

**Fig. 1:**
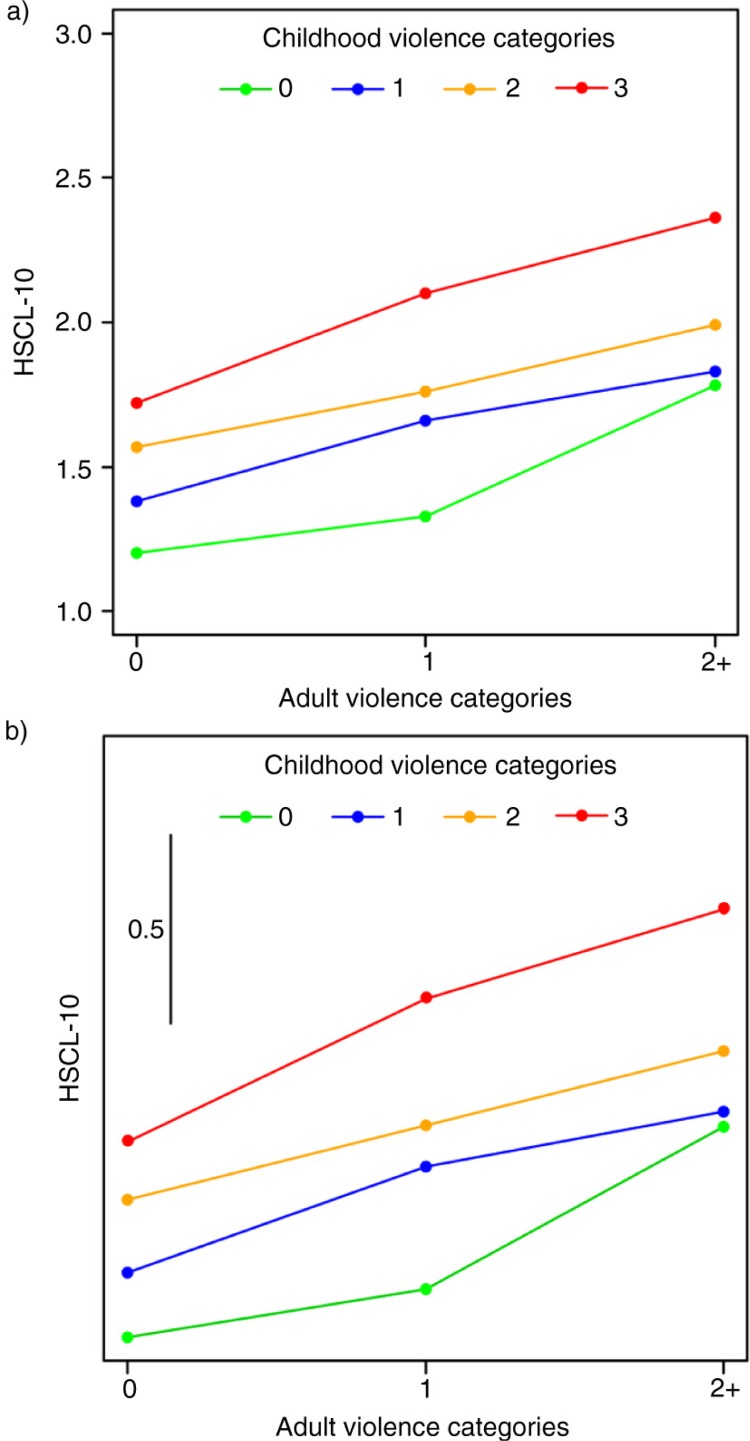
Unadjusted (a) and gender- and age-adjusted (b) associations between adult violence categories and psychological distress (HSCL-10) in groups exposed to zero, one, two, or three childhood violence categories.

Prevalence data were weighted for age and area of residence. The weights were constructed as inverse probability weights for the sample of responders based on population figures from Statistics, Norway. Table 1 presents unweighted and weighted data separately for women and men. Because only minor differences were found between weighted and unweighted prevalences, all tables and figures except Table 1 present unweighted data. Gender differences in violence exposure were tested with chi square statistics. In Tables 2–4 and Fig. 1, childhood violence was collapsed into broader categories: CSA and rape, before the age of 18 now represented “any childhood sexual abuse”; parental physical violence and parental IPV became “physical violence in the family”; and psychological violence, emotional neglect and physical neglect were collapsed into “psychological violence/neglect.” The relationship between childhood violence and adult violence was estimated by logistic regression analyses. Furthermore, we conducted a multiple linear regression analysis using the HSCL mean score as the outcome variable, adjusted for age and gender. Figure 1b displays the regression coefficients for the increase of the mean HSCL-10 associated with an increase in adult violence categories within each group of childhood violence categories. We used multiple linear regression analyses to investigate the association between HSCL-10 and all possible combinations of the three childhood violence exposure categories. All analyses were conducted in SPSS for Windows version 20.

**Table 1:** Lifetime prevalence of sexual abuse, physical abuse, psychological abuse, and neglect by gender

	Women			Men			
		
Violence categories	N	Unweighted%	Weighted%	N	Unweighted%	Weighted%	χ^2^ p value^b^
Childhood sexual abuse							
Sexual abuse before the age of 13^a^	248	10.2	10.7	74	3.5	3.6	<0.001
Forcible rape	113	4.7	4.6	19	0.9	0.9	<0.001
Childhood family violence							
Physical violence from caretaker	117	4.9	5.0	103	5.1	5.2	0.865
IPV between caretakers	240	9.9	9.6	208	10.0	9.8	0.904
Neglect/psychological violence							
Psychological violence from caretaker	374	15.4	14.6	233	11.2	11.0	<0.001
Emotional neglect	237	9.8	9.5	179	8.6	8.7	0.199
Physical neglect	133	5.5	5.2	98	4.7	4.7	0.245
Adult abuse							
Forcible rape	150	6.2	5.8	7	0.3	0.3	<0.001
Physical violence	147	6.1	5.6	285	13.7	13.5	<0.001
Intimate partner violence	224	9.2	9.1	40	1.9	1.9	<0.001

a With or without penetration.

b χ^2^ tests performed with unweighted data.

**Table 2:** Associations between childhood and adult violence exposure, odds ratios (OR), and 95% confidence intervals of OR

	Adult forcible rape		Adult physical violence		Adult IPV	
	
	Women	Men^a^	Women	Men	Women	Men
Childhood violence	OR (95% CI)	OR (95% CI)	OR (95% CI)	OR (95% CI)	OR (95% CI)	OR (95% CI)
Sexual abuse^b^	5.95 (4.20–8.44)	–	3.57 (2.46–5.19)	5.46 (3.49–8.56)	2.55 (1.84–3.54)	3.51 (1.34–9.21)
Violence in the family^c^	3.76 (2.60–5.43)	–	2.40 (1.61–3.57)	2.99 (2.19–4.08)	3.57 (2.60–4.92)	4.24 (2.16–8.30)
Neglect/psychological violence^d^	4.46 (3.18–6.26)	–	2.54 (1.79–3.60)	2.66 (2.00–3.54)	3.98 (2.99–5.29)	2.43 (1.24–4.76)

a n=19.

b Sexual abuse=CSA before the age of 13 and/or rape before the age of 18.

c Violence in the family=physical violence from parents and/or parental IPV.

d Neglect/psychological violence=emotional neglect, physical neglect and/or psychological violence. All these single items were significantly (p≤0.011) associated with all adult violence exposure variables, except the association between physical neglect and IPV for men and all associations with adult rape, which could not be tested due to low n.

**Table 3:** Psychological distress (HSCL-10) in various exposure groups

	Adult violence categories (0–2)					
	
	0 Adult abuse (n=3783)		1 adult abuse (n=645)		2 or more adult abuse (n=100)	
	
Childhood violence categories (0–3)	Mean HSCL	% above cut-off HSCL	Mean HSCL	% above cut-off HSCL	Mean HSCL	% above cut-off HSCL
0 (n=3270)	1.20	5.0	1.33	9.6	1.73	28.1
1 (n=670)	1.35	12.1	1.63	29.3	1.86	35.3
2 (n=306)	1.48	19.7	1.70	32.5	1.83	47.1
3 (n=282)	1.77	38.2	1.95	50.5	2.32	64.7

**Table 4:** Associations between various combinations of childhood violence and HSCL-10 adjusted for adult violence, gender and age

Independent variables	Regression coefficient	95% CI
*Childhood violence*		
1 Sexual abuse alone (n=167)	0.117	0.054–0.181
1 Violence in the family alone (n=201)	0.128	0.071–0.186
1 Neglect/psychological violence alone (n=384)	0.261	0.218–0.304
2 Sexual abuse and violence in the family (n=42)	0.171	0.045–0.297
2 Sexual abuse and neglect/psychological violence (n=87)	0.450	0.362–0.537
2 Violence in the family and neglect/psychological violence (n=225)	0.370	0.314–0.425
3 Sexual abuse, violence in the family and neglect/psychological violence (n=92)	0.637	0.550–0.724
*Adult violence*		
Forcible rape	0.241	0.170–0.312
Physical violence	0.188	0.146–0.230
IPV	0.188	0.133–0.243
*Demographics*		
Gender	0.070	0.045–0.095
Age	−0.001	−0.002–0.001

Univariate regression coefficient for child sexual abuse: 0.366, 95% CI=0.322–0.411; for physical violence in childhood family: 0.325, 95% CI=0.286–0.363; for neglect/psychological violence: 0.399, 95% CI=0.367–0.431; for adult forcible rape: 0.529, 95% CI=0.459–0.599; for adult physical violence: 0.265, 95% CI=0.221–0.309; for adult IPV: 0.380, 95% CI=0.325–0.435; for gender: 0.109, 95% CI=0.083–0.135; and for age: –0.001, 95% CI= –0.002–0.000; p<0.001 for all associations except age (p=0.014).

## Results

The sample comprised 2,437 women (53.8%) and 2,091 men (46.2%) and the mean age was 44.4 years (range=18–74). The majority of participants were currently married or cohabitating (64.5%), were working or studying (76.9%), and had a college or university education (52.3%).

Prevalences of childhood and adult violence exposures are displayed in Table 1. CSA reported in Table 1 included vaginal, oral or anal penetration or attempted penetration for 4.0% of the total sample of women and 1.5% of the total sample of men. Table 1 displays rape separately for childhood and adulthood. The lifetime prevalence of forcible rape was 9.4% for women and 1.1% for men.

There were significant associations between the various violence categories reported during childhood. Participants who confirmed any CSA more often reported physical violence from caretaker (exposed: 16.7%, non-exposed: 3.8%), parental IPV (exposed: 25.2%, non-exposed: 8.3%), psychological violence (exposed: 38.4%, non-exposed 10.8%), emotional neglect (exposed: 25.3%, non-exposed: 7.3%), and physical neglect (exposed: 15.9%, non-exposed: 3.9%). Those who confirmed physical violence from caretaker more often reported any CSA (exposed: 28.8%, non-exposed: 7.4%), parental IPV (exposed: 46.5%, non-exposed: 7.2%), psychological violence (exposed: 64.8%, non-exposed: 9.9%), emotional neglect (exposed: 49.1%, non-exposed: 6.6%), and physical neglect (exposed: 32.1%, non-exposed: 3.3%). Among those who had experienced psychological violence and/or neglect, 22.9% reported any CSA (5.9% in non-exposed), 20.7% reported physical violence from caretakers (1.6% in non-exposed), and 29.7% reported parental IPV (5.5% in non-exposed). For all these associations, χ^2^ p-values were <0.001.

For both men and women, there were strong and significant relationships between childhood violence and adulthood violence that was not restricted to violence within a similar category (Table 2). Childhood exposure was associated with a 2.2–5.0 times higher occurrence of adult violence. The highest overlap was observed for women reporting CSA and adult rape (20.3% adult rape in exposed versus 4.1% in non-exposed), followed by women reporting parental psychological violence/neglect and adult rape (15.3% in exposed versus 3.9% in non-exposed) and men reporting CSA and adult physical violence (43.5% in exposed versus 12.4% in non-exposed).

Anxiety/depression increased with the number of childhood and adult violence categories experienced (Table 3). Figure 1a illustrates the observed mean HSCL scores associated with adult exposure in individuals exposed to zero, one and two or more childhood violence categories. Anxiety/depression scores associated with adult abuse increased with the number of childhood violence categories. Figure 1b displays the results of a multiple regression analysis for the HSCL mean scores of adulthood abuse and childhood abuse and shows that anxiety/depression scores increased with both adult and childhood violence. Adult exposure was significantly associated with a higher HSCL mean score for all levels of childhood violence (p<0.001). There was a significant interaction effect between childhood violence and adult violence (p=0.012), which was due to a smaller increase in HSCL from zero to one adult violence category for those exposed to zero childhood violence (the green line in Fig. 1b).

Childhood violence exposure was significantly associated with adult anxiety/depression, even when adjusted for violence in adulthood (overall p-value<0.001, Table 4). Among participants exposed to one childhood violence category, those exposed to neglect and/or psychological violence reported more anxiety/depression than those exposed to sexual abuse alone (p<0.001) or family violence alone (p<0.001). Of those who were exposed to two childhood violence categories, those exposed to neglect/psychological violence in combination with sexual abuse and/or family violence reported more anxiety/depression compared to individuals reporting the combination of sexual abuse and family physical violence (p<0.001 for both comparisons). Individuals experiencing three childhood violence categories had the highest anxiety/depression scores (p≤0.007 for all comparisons). Adult violence was also uniquely associated with anxiety/depression.

## Discussion

A substantial proportion of the Norwegian population reported exposure to violence. Among women, for example, 9.4% reported that they had been victims of forcible rape at least once. This is higher than the 5% rape prevalence reported in a recent study of violence in 28 European countries (Violence against women: an EU-wide survey. Main results, 2014). However, that study used an unusually strict rape definition, and used face-to-face interviews, which is known to reduce the willingness to disclose sensitive information (Jansson, 2007). The rape prevalence found in the current study is in agreement with a newly published Swedish study (Nationellt Centrum för Kvinnofrid, 2014), which found that 11% of women older than 18 years experienced rape/attempted rape. It is also in agreement with a previous study from Denmark that reported a 9% lifetime rape in women (Balvig & Kyvsgaard, 2006), but somewhat lower than the 13% rape prevalence for women reported in the United States (Resnick et al., 1993). In our study, women carried a higher total burden of violence because they were more often exposed to sexual abuse and IPV than men.

Childhood victimisation was strongly associated with adult victimisation. This indicates a substantial risk of revictimisation in violence-exposed children, which is in accordance with previous retrospective and prospective studies (Koenen & Widom, 2009; Trickett, Noll, & Putnam, 2011). The current study expanded on previous knowledge by showing that the overlap between childhood and adult victimisation seemed to be unspecific; that is, any childhood victimisation was associated with any adult violence exposure. The associations were substantial, ranging from a two to five times higher occurrence of adult violence in exposed children. This finding contrasts with the previous main focus on CSA and sexual revictimisation (Classen et al., 2005) and concurs with recent calls for a broad research and prevention approach that targets all forms of violence against children (Finkelhor et al., 2007).

In line with previous research (Chapman et al., 2004; Dube et al., 2001; Gilbert et al., 2009), mental health problems showed a substantial and graded relationship to the number of childhood victimisation categories. Few studies have investigated both childhood and adult victimisation in detail, and our study adds to existing knowledge by showing that anxiety/depression associated with adult violence exposure increased systematically with increased childhood victimisation. All potential combinations of childhood violence were associated with anxiety/depression; however, psychological violence/neglect seemed to be particularly important. Although some studies have found CSA to be more damaging to mental health than other forms of violence (Widom, 1989), other studies have noted the importance of psychological violence and neglect (Gilbert et al., 2009; Norman et al., 2012; Teicher et al., 2006). Neglect and psychological violence may have a prominent impact on health because they are inherently long-lasting; in contrast, sexual and physical abuse are distinct events, although they may occur repeatedly. The combination of psychological violence/neglect and CSA or physical abuse seemed to be of particular importance for adult mental health. Our results concur with the increasingly large amount of literature finding that the burden of childhood violence may last a lifetime and underscore the long-term public health problems associated with violence against children.

Previous research has shown that revictimisation may be an important explanation for why violence-exposed children have increased mental health problems later in life (Koenen & Widom, 2009). This finding may not be due only to the increased risk of violence in adult life. Our study indicates that childhood violence makes individuals more vulnerable to suffering negative health consequences of the violence they experience in adulthood. Similar results have been found in a previous study (Koopman et al., 2005). Revictimisation and mental health are most likely interrelated, and previous research has also found mental health problems to be a risk factor for revictimisation—for example, through symptoms of PTSD (Arata, 2000; Ullman, Najdowski, & Filipas, 2009). Furthermore, complex relationships that include genetic factors, changes in stress-response systems, attachment, social support, and other environmental and individual conditions are also likely to play a role in revictimisation and mental health development (Pratchett & Yehuda, 2011). Prospective studies that investigate potential mediators and moderators are necessary to understand why, and for who, such negative development occurs, which will help to target prevention measures and improve care for victims.

## Limitations

The current study was cross-sectional; therefore we cannot make causal inferences. Memory for past events may be influenced by current states, such as an ongoing depression. Current depressed mood may lead individuals to interpret past events more negatively, which may have resulted in an overestimation of the associations between past violence exposure and current anxiety/depression. This may have been particularly the case for emotional neglect and psychological violence, as these measures are more subjective in nature, compared to the more behaviourally specific forms of physical and sexual abuse. Although the measures of neglect and psychological violence in this study resembles those used in several other studies (Centers for Disease Control and Prevention, 2014; Christoffersen, Armour, Lasgaard, & Elklit, 2013; Green et al., 2010; Kilpatrick et al., 2003), the questions were simple, and would not be expected to capture the full variety of these phenomena. Lack of sufficient parental care and psychological abuse can happen in many different ways, in various time periods in childhood, and may have differential effects depending on the developmental stage of the child. There is currently no common agreement on how these phenomena are best measured. Hence, psychological violence and neglect has received less research attention than physical violence and sexual abuse (Gilbert et al., 2009).

The majority of the sample from the General Population Registry for who we were able to identify a telephone number, never answered the phone, and 57% of people who we were able to contact rejected participation. The comparisons of participants to general population data suggested a positive selection of respondents in terms of education and income, which may indicate that our prevalence estimates of violence and abuse are somewhat conservative.

Individuals with abuse histories may have found the study more relevant for them, and may have been more willing to participate in the study. It is also possible that violence-exposed individuals may find it hard to talk about their experiences, which would result in an underestimation of abuse prevalences. Analyses within responders of number of calls necessary to get in contact did not support the hypothesis that exposed individuals made themselves more available (see Appendix 1). However, women with a history of parental physical violence and men with a history of emotional neglect were both slightly easier to contact, which might imply a small overrepresentation of some exposure groups. On the other hand, forgetfulness, denial, misunderstanding, and embarrassment may result in false-negative reports (Gilbert et al., 2009), which may lead to under-reporting rather than over-reporting of childhood abuse (Fergusson et al., 1997). We conclude that the presented prevalence rates should be interpreted with caution.

The relationships between variables would, presumably, suffer less from a biased sample. Childhood victimisation may be related to a range of mental and somatic health consequences, but this study included only symptoms of depression and anxiety. Strengths of the study include the large sample size, the remarkably low level of missing information among respondents, and the broad assessment of childhood and adult victimisation.

Our results suggest that more effort is needed to identify and assist victimized children and follow them over time, and are in support of the newly published NICE guidelines that recommend routine screening for violence (National Institute for Health and Care Excellence, 2014). Such efforts have the potential to substantially improve mental health and quality of life of the general population. Child clinicians should be aware that child victims of violence carry an increased risk for future victimisation. It is important to note that the increased revictimisation risk seems not to be restricted to the same type of violence. The combination of both childhood victimisation and adult revictimisation is associated with particularly severe levels of psychological distress in adulthood.

## Supplementary Material

Violence against children, later victimisation, and mental health: a cross-sectional study of the general Norwegian population

Violence against children, later victimisation, and mental health: a cross-sectional study of the general Norwegian population

Violence against children, later victimisation, and mental health: a cross-sectional study of the general Norwegian population

## Appendix 1

Analyses of associations between number of calls to get in touch and socio-demographic variables, exposure variables, and mental health.

Analyses of socio-demographic differences between responders and non-responders cannot tell us whether the sample is biased in the variables under investigation. It may be likely that individuals who were especially affected by the terrorist attacks considered the study to be more relevant to them, potentially resulting in an over-representation of affected individuals. We hypothesized that those individuals who were most interested in the study would, after receiving the invitation letter, make themselves more available by telephone, resulting in fewer calls necessary to get in touch, and that those who were “hard to contact” and required many calls, would look more like non-responders. Similar procedures have been used previously, as non-responders are believed to behave more like late responders (Danice, Jackson, & White, 2012; Thoresen, Aakvaag, Wentzel-Larsen, Dyb, & Hjemdal, 2012). We investigated the number of calls required to make contact and socio-demographic variables and violence exposure. We used Mann–Whitney U tests to analyse differences between groups in number of calls, and Pearson's correlation for the association between age and number of calls.

Socio-demographic variables: Somewhat fewer calls were necessary to make contact with women (mean number of calls=3.0, SD=2.0) compared to men (mean=3.1, SD=2.1), p=0.015. This gender difference was small, but statistically significant, and this is in agreement with the higher participation rate in females. Number of calls was also significantly correlated with age (r=−0.17, p<0.001), implying that fewer calls were necessary to reach older individuals. Marital status (p=0.714) and level of education (p=0.224) were not significantly associated with number of calls required. Individuals who were currently unemployed, retired, or for other reasons not working or studying however, needed fewer calls (mean=2.6, SD=1.7) compared to those who were currently working or studying (mean=3.2, SD=2.1), p<0.001. This finding may reflect higher availability in non-working groups.

Violence exposure and mental health: These analyses were conducted separately for women and men (Table 5). We found no significant differences in the number of calls necessary to reach those exposed versus not exposed for sexual contact before the age of 13; lifetime forcible rape; other sexual assaults; psychological violence; parental physical neglect; parental IPV; severe physical violence in adulthood or adult IPV. Fewer calls were necessary to reach women exposed to parental physical violence in childhood and men exposed to parental emotional neglect. These differences were small, but statistically significant. Number of calls was not significantly associated with anxiety/depression (HSCL-10; r=−0.085, p=0.494) or with posttraumatic stress symptoms (PCL-6; r=−0.01, p=0.494). To conclude, these “hard to contact” analyses do not generally support the hypothesis that individuals with more exposure or more mental health problems were easier to contact. However, women who reported physical violence in childhood and men who reported emotional neglect seemed to be slightly more available. This might indicate a small overrepresentation of these types of violence.

**Table 5:** Number of calls to reach respondent in relation to violence exposure

		Women			Men		
		
Violence exposure		Mean no of calls	SD	p	Mean no of calls	SD	p
Lifetime forcible rape	Exposed	3.0	2.1	0.875	3.7	2.6	0.348
	Not exposed	3.0	2.0		3.1	2.1	
Sexual contact before age 13	Exposed	3.0	2.0	0.896	3.1	1.7	0.579
	Not exposed	3.0	2.0		3.1	2.1	
Other sexual assaults lifetime	Exposed	3.1	2.1	0.308	3.2	2.0	0.391
	Not exposed	3.0	2.0		3.1	2.1	
Severe parental physical violence	Exposed	2.6	1.8	0.020	2.9	2.0	0.175
	Not exposed	3.0	2.1		3.2	2.1	
Parental psychological violence	Exposed	2.9	2.0	0.792	3.1	2.0	0.853
	Not exposed	3.0	2.1		3.2	2.1	
Parental IPV	Exposed	2.7	1.8	0.072	3.2	2.2	0.631
	Not exposed	3.0	2.1		3.1	2.1	
Parental emotional neglect	Exposed	2.8	1.9	0.055	3.2	2.1	0.029
	Not exposed	3.0	2.1		2.9	2.0	
Parental physical neglect	Exposed	2.8	1.9	0.396	3.0	2.0	0.657
	Not exposed	3.0	2.0		3.2	2.1	
Severe physical violence in adulthood	Exposed	3.0	2.1	0.708	3.2	2.1	0.130
	Not exposed	3.0	2.0		3.1	2.1	
Adult IPV	Exposed	3.1	1.9	0.158	3.1	2.0	0.860
	Not exposed	3.0	2.1		3.1	2.1	

Mann–Whitney U tests.
