# Supplementary material for: Violence against children, later victimisation, and mental health: a cross-sectional study of the general Norwegian population
Source: Eur J Psychotraumatol. 2015 Jan 13;6:10.3402/ejpt.v6.26259. doi: 10.3402/ejpt.v6.26259 (PMC4296052; doi:10.3402/ejpt.v6.26259)
Supplement: Violence against children, later victimisation, and mental health: a cross-sectional study of the general Norwegian population [file EJPT-6-26259-s001.pdf]

Çocuklara karşı şiddet, ilerideki viktimizasyon ve ruh sağlığı: Genel (ülke) nüfusta enlemesine bir çalışma

Siri Thoresen, Mia Cathrine Myhre, Tore Wentzel-Larsen, Helene Flood Aakvaag, Ole Kristian Hjemdal

Özet
Arkaplan: Çocukluktaki şiddet ruh sağlığı sorunları ve yinelenen viktimizasyonla ilişkilidir. Çocukluk cağı ve yetişkinlikteki farklı türlerdeki viktimizasyonun yetişkin ruh sağlığı üzerindeki göreceli önemi hakkında yeterli bilgi yoktur.
Yöntem: Araştırma, Norveç'teki yetişkin nüfusta enlemsel bir telefon görüşmesi çalışmasıdır. 18-75 yaş arası 2435 kadın ve 2092 erkek katılmıştır (aramaya çalıştıklarımızın %19,3'ü ve telefona cevap verenlerin %42,9'udur). Görüşme, hem çocukluk çağı hem yetişkinlikteki şiddete maruz kalmayı geniş yelpazeli bir şekilde içermektedir. Kaygı/depresyon Hopkins Belirti Kontrol Listesi (Hopkins Symptom Check List, HSCL-10) ile ölçülmüştür.
Sonuç: Viktimizasyon çok sık rapor edilmektedir; örneğin çocuk cinsel istismarı (kadın : %10,2, erkek: %3,5), çocukluk cağı ebeveyn şiddeti (kadın: %4,9, erkek: %5,1) ve yaşam boyu cebri tecavüz (kadın: %9,4, erkek: %1,1). Bütün çocukluk çağı şiddet türleri yetişkinlikteki viktimizasyon ile anlamlı bir bicimde ilişkilidir, maruz kalan çocuklarda 2,2 - 5 kere daha fazla meydana gelmiştir ($p < 0.05$ bütün ilişkiler için). Çocukluk çağında maruz kalınan şiddet türlerinin sayısı arttıkça, yetişkinlik çağı istismarı ile ilintili kaygı/depresyon (HSCL-10) artmaktadır ($p < 0.001$). Bütün çocukluk çağı şiddet kombinasyonları kaygı/depresyon ile ilişkilidir ($p < 0.001$ bütün ilişkiler için). En yüksek kaygı/depresyon seviyeleri psikolojik şiddet/ihmal rapor eden bireylerde görülmüştür.
Tartışma: Sonuçlar düşük yanıt oranının ışığında yorumlanmalıdır. Çocukluk çağı şiddetinin bütün türleri yetişkinlikteki viktimizasyon için bir risk faktörüdür. Yetişkinlikteki kaygı/depresyon hem deneyimlenen şiddet türlerinin sayısı hem de tipiyle ilişkilidir. Hem araştırma hem de önleme çalışmaları için çocukluk çağında ve yetişkinlikte şiddete maruz kalma geniş çaplı bir şekilde değerlendirilmelidir. Psikolojik şiddet ve ihmal, özellikle diğer şiddet türleriyle ile birlikte araştırmalarda daha çok yer bulmalıdır

Anahtar kelimeler: şiddet; çocuk istismarı; çocuk cinsel istismarı; tecavüz; ruh sağlığı; yinelenen viktimizasyon; epidemiyoloji; kaygı; depresyon

Name of translator: Emek Yuce Zeyrek-Rios

Citation: European Journal of Psychotraumatology 2015, 6: 26259 - http://dx.doi.org/10.3402/ejpt.v6.26259
