# Supplementary material for: Violence against children, later victimisation, and mental health: a cross-sectional study of the general Norwegian population
Source: Eur J Psychotraumatol. 2015 Jan 13;6:10.3402/ejpt.v6.26259. doi: 10.3402/ejpt.v6.26259 (PMC4296052; doi:10.3402/ejpt.v6.26259)
Supplement: Violence against children, later victimisation, and mental health: a cross-sectional study of the general Norwegian population [file EJPT-6-26259-s002.pdf]

Siri Thoresen, Mia Cathrine Myhre, Tore Wentzel-Larsen, Helene Flood Aakvaag, Ole Kristian Hjemdal

Abstract
Przemoc w dzieciństwie, późniejsza wiktymizacja oraz zdrowie psychiczne: badanie poprzeczne w populacji ogónej.
Wprowadzenie: Przemoc doświadczona w dzieciństwie jest związana z problemami ze zdrowiem psychicznym i ryzykiem rewiktymizacji. Niewiele natomiast wiadomo na temat znaczenia różnych form wiktymizacji w dzieciństwie i w dorosłości na zdrowie psychiczne wśród dorosłych.
Cel: Celem niniejszej pracy było okreśelnie związku pomiedzy różnymi formami wiktymizacji w dzieciństwie i w dorosłości na zdrowia psychiczne w dorosłości.
Metoda: Niniejsze badanie utrzymane było w schemacie poprzecznym i polegało na przeprowadzeniu ankiety telefonicznej wsród populacji dorosłych Norwegów (2435 kobiet i 2092 mężczyzn w wieku 18-75 lat) na temat doświadczenia szerokiej gamy ekspozycji an przemoc w dzieciństwie i w dorosłości. Lęk i depresję mierzono za pomocą kwestionariusza Hopkins Symptom Check List (HSCL-10).
Wyniki: Odnotowano wysoki procent doświadczenia różnych form wiktymizacji wśród osób badanych: wykorzystywanie seksulane (10.2% kobiet i 3,5% mężczyzn), przemoc fizyczna w dzieciństwie (4,9% kobiet i 5,1% mężczyzn), gwałt (9,4% kobiet i 1,1% mężczyzn). Wszystkie kategorie przemocy w dzieciństwie były istotnie związane z wiktymizacją w dorosłości. Lęk/depresja były związane z wykorzystaniem w dorosłości, natomiast przejawianie wysokiego poziomu przemocy/wykorzystania w dzieciństwie było powiązane z poziome lęku i depresji.
Konkluzje: Przemoc w dzieciństwie była czynnkime ryzyka wiktymizacji w dorosłości. Lek/depresja były istotnie związane ze wszystkimi kategoriami przemocy.
Słwoa kluczowe: Przemoc; wykorzystanie w dzieciństwie; wykorzystanie seksualne w dzieciństwie; gwałt; zdrowie psychiczne; rewiktymizacja; epidemiologia; lek, depresja.

Name of translator: Marcin Rzeszutek, University of Finance and Management in Warsaw, Poland

Citation: European Journal of Psychotraumatology 2015, 6: 26259 - http://dx.doi.org/10.3402/ejpt.v6.26259
