# Supplementary material for: Violence against children, later victimisation, and mental health: a cross-sectional study of the general Norwegian population
Source: Eur J Psychotraumatol. 2015 Jan 13;6:10.3402/ejpt.v6.26259. doi: 10.3402/ejpt.v6.26259 (PMC4296052; doi:10.3402/ejpt.v6.26259)
Supplement: Violence against children, later victimisation, and mental health: a cross-sectional study of the general Norwegian population [file EJPT-6-26259-s003.pdf]

Gewalt an Kindern, spätere Viktimisierung und psychische Gesundheit: Eine Querschnittsstudie der allgemeinen (Land-) Bevölkerung

Siri Thoresen, Mia Cathrine Myhre, Tore Wentzel-Larsen, Helene Flood Aakvaag, Ole Kristian Hjemdal

Hintergrund: Gewalt in der Kindheit ist verbunden mit gesundheitlichen Problemen und dem Risiko der Reviktimisierung. Es ist wenig bekannt über die Bedeutung der verschiedenen Typen von kindheitlicher Viktimisierung und solcher bei Erwachsenen in Bezug auf die Gesundheit im Erwachsenenalter.

Ziel: Ziel dieser Studie war es, die Zusammenhänge zwischen den verschiedenen Typen von Gewalt in der Kindheit und im Erwachsenenalter aufzuzeigen, sowie deren Zusammenhang mit Gesundheit im Erwachsenenalter.

Methode: Diese Studie wurde als telefonische Querschnittsstudie an norwegischen Erwachsenen durchgeführt. 2435 Frauen und 2092 Männer im Alter von 18-75 Jahren nahmen teil (19.3% versuchten wir telefonisch zu erreichen, 42.9% nahmen das Telefonat an). Das Interview bestand aus einer großen Darstellung der Gewalt in der Kindheit wie auch im Erwachsenenalter. Angst und Depression wurde mittels der Hopkins Symptom Check List (HSCL-10) gemessen.

Ergebnisse: Es wurde häufig von Viktimisierung berichtet, wie z.B. sexueller Missbrauch von Kindern (Frauen: 10.2%, Männer 3.5%), physische Gewalt durch Eltern in der Kindheit (Frauen: 4.9%, Männer: 5.1%), und schwere Vergewaltigung über die Lebensspanne (Frauen: 9.4%, Männer: 1.1%). Alle Kategorien von Gewalt in der Kindheit waren signifikant mit Viktimisierung im Erwachsenenalter verbunden, mit einer 2.2 bis zu 5 mal höherer Häufigkeit bei gefährdeten Kindern ($p<0.05$ für alle Gruppen). Angst/Depression (HSCL-10) im Zusammenhang mit Missbrauch im Erwachsenenalter stieg mit der Anzahl von erlebten kindheitlichen Gewaltakten ($p<0.001$). Alle Ausprägungen von kindheitlicher Gewalt waren signifikant mit Angst und Depression verknüpft ($p<0.001$ für alle Gruppen). Bei berichteter psychischer Gewalt/Vernachlässigung zeigten sich die höchsten Raten von Angst und Depression.

Schlussfolgerung: Bei der Interpretation der Ergebnisse ist stets die geringe Rücklaufquote zu berücksichtigen. Gewalterfahrung in der Kindheit in all ihren Formen war ein Risikofaktor für die Viktimisierung im Erwachsenenalter. Angst und Depression im Erwachsenenalter war mit der Anzahl der Gewaltkategorien und der Art der erlebten kindheitlichen Gewalt verbunden.
Für Forschungs- und Präventionszwecke ist eine umfassende Bewertung von Gefährdung und Gewalt in der Kindheit und im Erwachsenenalter notwendig. Es sollte ein größeres Augenmerk in der Forschung auf psychische Gewalt und Vernachlässigung, vor allem in Kombination mit anderen Arten von Gewalt, gelegt werden.

Keywords/Schlagwörter: Gewalt, Kindesmissbrauch, sexueller Missbrauch von Kindern, Vergewaltigung, geistige Gesundheit, Reviktimisierung, Epidemiologie, Angst, Depression

Name of translator: Iris Fischer, Brigitte Lueger-Schuster

Citation: European Journal of Psychotraumatology 2015, 6: 26259 - http://dx.doi.org/10.3402/ejpt.v6.26259
